# Modification of the pneumatic retinopexy for the treatment of rhegmatogenous retinal detachment with multiple-quadrant retinal breaks

**DOI:** 10.3389/fmed.2025.1549152

**Published:** 2025-03-12

**Authors:** Xin Zhang, Haixiu Wu, Shenchao Guo, Tiepei Zhu, Zhenyang Xiang, Gaochun Li, Enhui Li

**Affiliations:** ^1^Department of Ophthalmology, Taizhou Hospital of Zhejiang Province Affiliated to Wenzhou Medical University, Taizhou, China; ^2^Eye Center, Second Affiliated Hospital of Medical College, Zhejiang University, Hangzhou, China

**Keywords:** gas tamponade, laser photocoagulation, pneumatic retinopexy, rhegmatogenous retinal detachment, retinal break

## Abstract

**Purpose:**

This study aimed to evaluate the efficacy of a modified pneumatic retinopexy (PR) procedure for patients with rhegmatogenous retinal detachment (RRD), particularly those with multiple-quadrant retinal breaks that spanning more than three clock-hours of retinal arc.

**Methods:**

This prospective case series included ten eyes of ten patients, all of whom underwent the PR surgery during hospitalization. The modified PR technique involved an intravitreal injection of a low concentration of perfluoropropane (14% C_3_F_8_) for gas tamponade and head positioning maneuvers guided by an indicator. The multiple breaks were closed by gas bubble and treated with immediate laser photocoagulation one by one during the sequential alternating head positioning maneuver.

**Results:**

After the modified PR surgery, retinal attachment was achieved in 9 of the 10 eyes (90%). The single case of failure, due to significant vitreous hemorrhage following gas injection, underwent a vitrectomy and received retinal attachment. Permanent retinal attachment was achieved in all cases (100%) at the last follow-up with no adverse events. The mean preoperative best-corrected visual acuity was improved significantly at 6 months post-surgery.

**Conclusion:**

This study demonstrates the potential of modified PR to achieve high success rates in RRD cases with multiple-quadrant retinal breaks cases involving superior retinal detachments and early proliferative vitreoretinopathy.

## Introduction

Emerging epidemiological data suggest a gradual escalation in the incidence of rhegmatogenous retinal detachment (RRD) requiring surgical intervention, with projections indicating a potential doubling of current prevalence rates within the coming two decades (1–4). Pneumatic retinopexy (PR) is a minimally invasive technique for repairing RRD, primarily suitable for uncomplicated cases with limited superior retinal breaks (5–10). Traditionally performed in an office setting, the success of PR is highly dependent on patient compliance with postoperative head positioning (11–13). Conventional methods typically involve injecting expansion gasses like 100% C_3_F_8_ or SF_6_, which, despite their effectiveness, have drawbacks such as slow onset and prolonged retention in the vitreous cavity, leading to increased risks of new retinal breaks and proliferative vitreoretinopathy (PVR) (14, 15).

In our previous research, we demonstrated a modified PR technique using pure air for intravitreal injection, enabling rapid retinal repositioning and supplemented by hospitalization for close monitoring and prompt laser photocoagulation (16). We also presented a modified PR approach utilizing a low concentration of expansile gas and a head position maneuver, demonstrating its success in treating a patient with simultaneous bilateral RRD (17). This approach yielded high reattachment rates in simple RRD cases. However, RRD involving multiple-quadrant breaks presents significant challenges for both traditional and modified PR, resulting in lower surgical success rates, often necessitating vitrectomy as an alternative treatment. Vitrectomy, while effective for complex detachments, is more invasive and carries risks such as cataract development, and the potential need for subsequent silicone oil removal (5). Additionally, studies have indicated increased rates of retinal displacement and loss of macular outer retinal layers compared to PR (18, 19).

To broaden the indications for PR, this study explores a novel modification that employs low-concentration expansion gas (14% C_3_F_8_) in conjunction with a head positioning maneuver. Our aim is to assess the efficacy of this approach in treating RRD cases with multiple-quadrant retinal breaks.

## Materials and methods

This study was a single-center prospective case series, patients who had complex RRD with multiple breaks were included. The following were the inclusion criteria: Patients with retinal detachment primarily caused by horse-shoe tears. Fresh retinal detachment with superior breaks located range from 8 to 4 o’clock, and with multiple breaks separated by more than 3 o’clocks. Exclusion criteria include inferior breaks between 4 to 8 clock hours in detached retina, or with PVR grade C or D, or with significant cloudy media, giant retinal tear or dialyses, or had a history of prior retinal surgeries. The study was approved by Taizhou Hospital Ethics Committee for Human Research and performed in accordance with the tenets of the Declaration of Helsinki.

### Intraocular gas volume

For retinal breaks separated by at least three clock hours, a gas volume capable of covering 120° retina is required to fully cover the retinal breaks. The literature describes that 25% of the vitreous cavity volume can cover 118° of the retina (20). However, for an emmetropic eye (with a vitreous cavity volume of approximately 4.5 mL), a gas volume of 1.15 mL is required. A large gas volume can significantly elevate the intraocular pressure (IOP) of the patient. In our approach, we use a specific head position maneuver to tamponade the retinal holes, which does not require such a large gas volume.

Theoretically, 0.1 mL of C3F8 gas can expand to its maximum volume of 0.44 mL on day 7.6 and then decay to 0.22 mL on day 13.3 (concurrently, air will dissipate around day 4) (21).Therefore, following a small clinical trial (including both emmetropic and highly myopic eyes) in several patients, and considering the volume of anterior chamber fluid we release and the intraocular pressure of the patients, we use a mixture of 0.1 mL C3F8 and 0.6 mL air for our surgery. We did not adjust the gas injection volume based on the axial length of the eye in this clinical trial.

### Surgical technique

Here, we present our modification of the PR technique for the treatment of complex RRD cases with multiple breaks separated by more than 3 o’clock. All procedures were conducted in a hospitalization setting. Super wide-angle fundus photography (Mirante, Nidex) was employed to document the fundus status both preoperatively and postoperatively.

**Figure 1:**
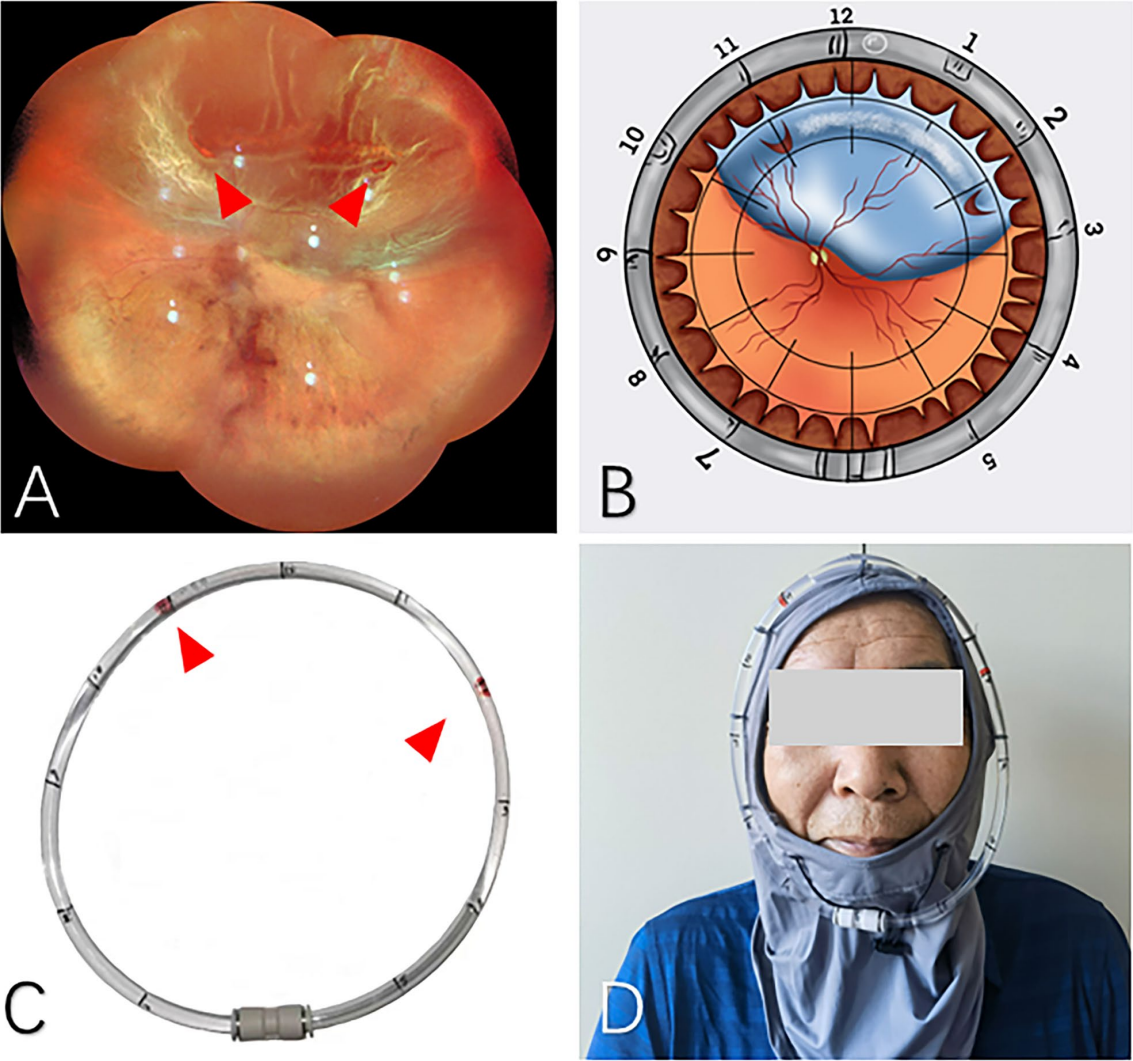
The application of head position indicator before PR treatment. **(A)** Super wide-angle fundus photography was applied to identify the retinal breaks preoperatively (red arrows). **(B)** Schematic diagram demonstrates the locations of the breaks. **(C)** The clock positions of retinal breaks were marked on the head position indicator (red arrows). **(D)** The patient could wear the head position indicator after intravitreal gas injection.

Unlike the traditional PR procedure, we utilized a low concentration perfluoropropane (14%, a mixture of pure air and C_3_F_8_) for the intravitreal injection to achieve a relatively long-lasting gas tamponade effect. Prior to gas injection, anterior chamber paracentesis was performed, and gentle pressure was applied using a microforceps to facilitate slow aqueous drainage. Following the gas injection, the patient was instructed to maintain a face-down position for 1 hour to protect the macula. Subsequently, the steam-roller technique was employed to reduce subretinal fluid. After the initial prone position, the patient needs to gradually adjust the head position. The patient will elevate the head by 30 degrees every hour until the target position is reached. By adjusting the position, the buoyancy of the gas bubble is utilized to expel the subretinal fluid through the retinal break into the vitreous cavity, reducing the subretinal fluid. A specific head position maneuver was then performed according to the preoperative locations of multiple retinal breaks in detached retina. The patient’s head position was adjusted to allow the gas bubble to exert pressure on the largest retinal break. Typically, the detached retina around this break reattached within 6–8 h, at which point retinal laser photocoagulation was applied to achieve 3–4 rows of laser spots around the break. If subretinal fluid persisted on one side of the break and laser treatment was not feasible due to ongoing retinal detachment, the head position was adjusted to move the gas bubble toward the side with subretinal fluid, thereby preventing the entry of liquefied vitreous into the subretinal space through the retinal break. After completing retinal laser treatment around the larger break, the head position can be further adjusted to allow the remained gas bubble to address additional retinal breaks. Similarly, immediate retinal laser photocoagulation could be applied when the retina is attached. Alternating head positioning on the left and right sides continued for 5 days until robust retina-choroidal adhesion was established. (see Figure 1 and Supplementary Video, which demonstrates the modified technique).

Additionally, we designed a head positioning indicator to assist in maintaining the correct head position (Figure 1). Prior to the PR surgery, the patient dons the head strap and maintains an upright position, allowing the surgeon to mark the corresponding clock positions of the retinal breaks on the plastic ring based on preoperative fundus photographs. During the post-injection head position maneuver, the patient’s head position can be adjusted to move the air bubble within the plastic ring to the previously marked positions, thereby ensuring that the intraocular gas bubble is accurately placed over the retinal break.

### Head position indicator

Head Position Indicator (HPI) stably fixed the locator on the periphery of the patient’s frontal face through the head fixator. We use the air bubbles in an arc-shaped locator (an arc-shaped aqueduct) to simulate the longitude of the bubbles in the eye when the patient is lying on his side, which was convenient for the patient to maintain an accurate position and conducive to retinal reattachment (Figure 1).When the HPI is on, the arc-shaped locator parallel to the equator of the eyeball, the locator was marked according to the location of the retinal break as suggested by the wide-field fundus photograph, so the location of the bubbles in the eye is indicated by the bubble in the arc-shaped locator. If the patient’s position changes, it can be reset in time. After the high-priority breaks were closed, additional breaks were marked and closed sequentially.

### Participant informed consent

The possible benefits and risks of PR treatment were explained to the patients and informed consent was obtained from all adult patients and from the parents if the patient was younger than 18 years old, in accordance with the Helsinki Declaration, before inclusion in the study. Institutional review board approval (X20210501) for this study was obtained from the Ethics Committee of Taizhou Hospital.

### Statistical analysis

Statistical analyses were performed using Stata 9 (College Station, TX) and Microsoft Excel (Redmond, WA). Statistical analyses was performed using Paired t-test by IBM SPSS Statistics 24.0. *p* < 0.05 were considered statistically significant.

## Results

Ten eyes of 10 consecutive patients with RRD meeting inclusion criteria were prospectively studied. The cohort comprised three women (30%) and seven men (70%), with a mean age of 52.3 ± 7.47 years (range, 33–58). The mean symptom duration was 3.60 ± 2.22 days (range, 1–7 days). Preoperative characteristics are detailed in Table 1. Each eye had an average of 3.60 ± 1.07 identified retinal breaks (range, 2–5), spanning 3 to 5 clock-hours, with breaks averaging 3.20 ± 0.42 h apart (range, 3–4). The intraocular pressure of the affected eyes ranged from 18 to 35 mmHg at 2 h postoperatively, and on the first postoperative day and at the last follow-up visit IOP were within the normal range.

**Table 1:** Preoperative clinical characteristics of patients.

Patient	Age	Sex	Duration of symptoms (day)	Lens status	PVD	Lattice degeneration	Numbers of break	Type of breaks	Clock hours of detached retina (hours)	Macula	PVR
1	58	Female	4	phakic	3		4	Horse shoe	4	On	B
2	54	Female	1	phakic	3	exist	5	Horse shoe and atrophic	5	Detached	B
3	58	Male	3	phakic	3	exist	2	Horse shoe	6	Detached	B
4	57	Male	1	phakic	3	exist	3	Horse shoe	3	Detached	B
5	56	Male	4	phakic	3	exist	3	Horse shoe and atrophic	4	Detached	A
6	54	Female	5	phakic	3	exist	2	Horse shoe	4	Detached	A
7	51	Male	2	phakic	2	exist	4	Horse shoe	5	On	A
8	54	Male	7	phakic	3		4	Horse shoe	5	Detached	B
9	33	Male	2	phakic	1	exist	4	Horse shoe and atrophic	3	Detached	B
10	48	Male	7	phakic	2	exist	5	Horse shoe	4	detached	B

Stages of posterior vitreous detachment: (1) Perifoveal separation with adhesion of vitreous to the fovea; (2) Complete separation of vitreous from the macula; (3) Extensive vitreous separation with adhesion of vitreous to the disc; (4) Extensive vitreous separation with adhesion of vitreous to the disc.

**Figure 2:**
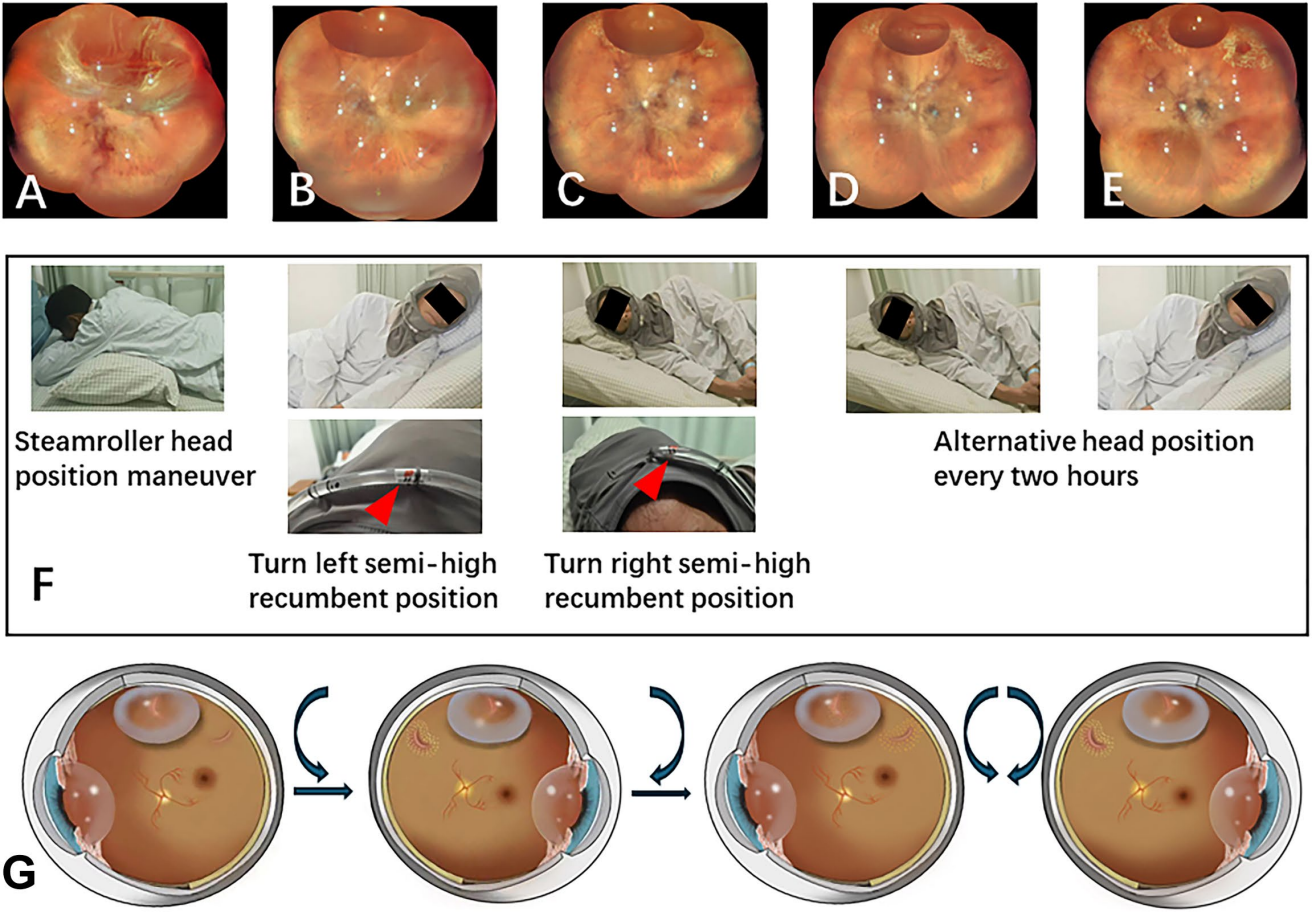
PR treatment after intravitreal gas injection. **(A)** The patient presented with lattice degeneration from 11 o’clock to 2 o’clock, accompanied by horseshoe retinal breaks at both ends of the degeneration. **(B)** Day 1 post-gas injection: After intravitreal gas injection, 3 h of face-down and steam roller position were executed, the patient was then instructed to remain in a left semi-high recumbent position to manage the retinal break at 11 o’clock all night long. Superior subretinal fluid was markedly reduced on the first postoperative day of examination. The nasal retina was reattached, Laser retinopexy was performed immediately on the retinal break at 11 o’clock and on the lattice degeneration under the gas bubble. Then the patient was then repositioned to the right semi-high recumbent position to address the retinal break at 2 o’clock. **(C)** Day 2 post-gas injection: laser retinopexy was performed on the retinal break at 2 o’clock and on the lattice degeneration under the gas bubble. **(D)** Day 3 post-gas injection: laser retinopexy was successfully strengthened for both retinal breaks. Patients alternate between these two positions every 2 hours. **(E)** Day 7 post-gas injection: the gas bubble had diminished, allowing for reinforcement of the laser retinopexy. **(F)** Illustration of the patient’s head position maneuver with the aid of the head position indicator during hospitalization. **(G)** The schematic diagram illustrates the sequential treatment of retinal breaks with a gas bubble, followed by continued alternative blockage by the gas bubble after the retina has been successfully reattached.

**Figure 3:**
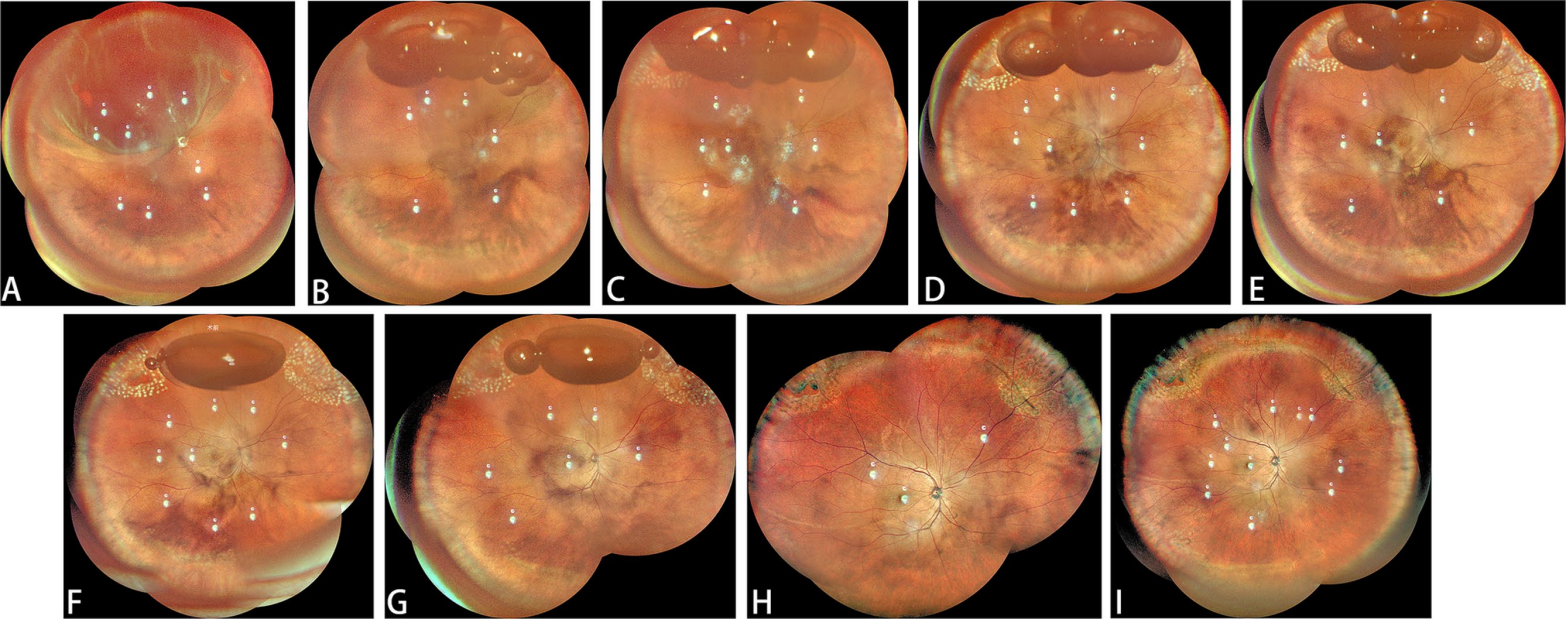
Another classic case. **(A)** The patient presented with lattice degeneration from 10 o’clock to 2 o’clock accompanied by horseshoe retinal breaks at both ends of the degeneration. **(B)** Day 1 post-gas injection: After intravitreal gas injection, before laser treatment. **(C)** Day 1 after laser treatment. **(D)** Day 2 post-gas injection: after first laser treatment in the morning. **(E)** Day 2 post-gas injection: after first laser treatment in the afternoon. **(F)** Day 3 post-gas injection. **(G)** Day 4 post-gas injection. **(H)** One month after surgery. **(I)** Six months after surgery.

Following modified PR, retinal attachment was achieved in 9 of the 10 eyes (90%), with all patients receiving only a single 14% C_3_F_8_ gas injection. No major intraoperative complication was noted, except for one eye that experienced significant vitreous hemorrhage following intravitreal gas injection. The PR failed eye was successfully managed with vitrectomy. All eyes attained permanent attachment by mean follow-up of 9.90 ± 4.68 months (range, 6–16 months). The mean preoperative BCVA (logarithm of the minimal angle of resolution) was 0.94 ± 0.71 (Snellen equivalent: 20/20 to 20/800), which improved significantly to 0.59 ± 0.39 at the 6 months follow-up (*p* < 0.05, *n* = 10). At the time of discharge, all patients had sufficient gas to tamponade more than one-clock-hour retinal arc. At the two-week follow-up visit, eight patients still exhibited very small air bubbles in their eyes. Two illustrative cases are shown in Figures 2, 3.

## Discussion

A synthesis of prior clinical studies reveals a median primary success rate of 69% (range: 51–90%) for pneumatic retinopexy (PR) procedures (22). This observed heterogeneity in surgical outcomes appears multifactorial, potentially attributable to variations in patient selection criteria, intraoperative surgical protocols, postoperative management strategies, implementation modalities of adjunctive cryotherapy compared with laser photocoagulation techniques, and differential application of gas types with varying injection volumes (11, 12, 23–26).

In the PR procedure, an ideal gas bubble should meet several criteria: first, the initial volume of gas bubble must be sufficient to cover the retinal breaks, facilitating early retinal reattachment and enabling prompt retinal photocoagulation. Second, during the formation of retinal-choroidal adhesion, the tamponade effect of the bubble should sustain the retina in a reattached position. Third, after achieving firm retinal-choroidal adhesion around the break (within 1–2 weeks), the gas bubble should gradually dissipate to minimize interference with the vitreous and retina (27). In this study, we utilized 14% C_3_F_8_, which offers the advantage of maintaining a relatively stable early gas volume compared to traditional pure C_3_F_8_, which is characterized by an initial expansion followed by contraction. Research indicates that a 1.2 mL gas bubble is required to adequately cover 80–90 degrees of the retina (28). Consequently, the initial injection of 0.6–0.7 mL of 14% C_3_F_8_ is often insufficient for tamponading multiple retinal breaks separated by more than 3 hours. Therefore, multiple breaks must be treated sequentially with the gas bubble. Additionally, the lower C_3_F_8_ concentration results in shorter gas presence (half-life approximately 10 days vs. 20 days for pure C_3_F_8_) (29), reducing vitreous traction and preventing new break formation.

Accurate identification and localization of all retinal breaks is a crucial principle in RRD surgeries. To prevent missing retinal breaks, super wide-angle fundus photography is used preoperatively. These images create clock-hour marks on a head positioning indicator, guiding post-intravitreal gas injection head positioning. This tool simulates the intraocular gas bubble’s position, aiding in precise head positioning to cover retinal breaks effectively. It is particularly useful in RRD cases with multiple-quadrant breaks, allowing the physician to address each break sequentially and reposition the patient’s head as needed for optimal tamponade. Additionally, if subretinal fluid persists after gas tamponade, the indicator helps adjust positioning to better occlude the break, preventing liquefied vitreous migration into the subretinal space and facilitating fluid absorption for subsequent laser treatment.

In managing multiple-quadrant retinal breaks, we employ a sequential alternating head positioning strategy to address each break individually. This approach prioritizes sealing larger breaks first due to the diminishing size of the intraocular gas bubble over time. When breaks are similar in size, we prioritize nasal or temporal breaks before superior breaks, as maintaining head position for superior breaks is generally easier. Following retinal laser treatment, the remaining gas bubble is utilized for periodic head position changes every 2 h, aligning it with the corresponding retinal breaks. This regimen ensures effective tamponade and enhances patient comfort and compliance with positioning protocols, promoting choroidal-retinal adhesion and facilitating retinal reattachment.

Vitreous hemorrhage is a common complication following PR (30). Jung et al. reported an incidence of 11.8%, rising to 20.0% in non-traditional PR cases (31). Our only instance of PR failure was due to vitreous hemorrhage, exacerbated by the complex nature of non-traditional cases involving multiple retinal breaks, mild pre-existing hemorrhage, and increased bleeding risk from intraocular gas movement. However, timely vitrectomy intervention as a remedial treatment did not compromise final retinal reattachment.

In the treatment of complex retinal detachment, pars plana vitrectomy (PPV) is generally prioritized over pneumatic retinopexy (PR) surgery. However, pneumatic retinopexy (PR) still retains its own advantages. In terms of anatomical reattachment, the PIVOT study reported that the success rate of retinal reattachment after PPV (93.1%) was significantly higher than that after PR (80.8%). However, after secondary surgical interventions, the final retinal reattachment rates in both groups exceeded 98%, with no significant differences. For cases where PR failed, there was no increased difficulty in subsequent PPV treatment, and patients achieved good visual recovery (Snellen acuity >20/40). Additionally, the PIVOT study found that 81% of patients undergoing PPV experienced cataract progression, with 65% requiring subsequent cataract surgery, compared to only 29% in the PR group (5). Considering that PR is less invasive and more cost-effective than PPV (32–34), and that PR outperforms PPV in terms of axial length change, retinal reattachment, retinal structural integrity, and visual recovery (18, 19, 35–40), the author suggests that PR should be the preferred treatment for non-inferior retinal detachment cases, achieving good anatomical outcomes and satisfactory visual function. For complex retinal detachment cases, such as those with media opacity (e.g., peripheral capsular opacification in pseudophakic eyes, dense vitreous hemorrhage), significant vitreoretinal proliferation, or inferior retinal breaks, PPV is the preferred treatment option.

While our preliminary results are promising, the limited number of participants restricts the statistical power and generalizability of our findings. To further evaluate the impact of the small sample size, we calculated the power based on the observed effect size (Cohen’s d = 0.8) and a significance level of 0.05. The analysis revealed that our study had a power of 65%, which is below the conventional threshold of 80%. This indicates that our sample size may have been insufficient to detect small to moderate effects, and the results should be interpreted with caution, and larger, multicenter studies with adequately powered sample sizes are needed to validate our findings.

While our study demonstrates favorable short-term outcomes with the modified PR technique, the mean follow-up period of 9.90 ± 4.68 months is insufficient to evaluate long-term complications such as PVR, recurrent retinal detachment. Future studies with extended follow-up periods are needed to fully validate the long-term efficacy of this technique.

In summary, our modified PR technique using low-concentration C3F8 gas and guided head positioning maneuvers has shown promising success in treating RRD with multiple-quadrant breaks involving superior retinal detachments and early proliferative vitreoretinopathy. However, due to our study’s small sample size, further investigation is needed to validate these outcomes.

## Data Availability

The original contributions presented in the study are included in the article/Supplementary material, further inquiries can be directed to the corresponding author.
